# Recognition of Morphometric Vertebral Fractures by Artificial Neural Networks: Analysis from GISMO Lombardia Database

**DOI:** 10.1371/journal.pone.0027277

**Published:** 2011-11-04

**Authors:** Cristina Eller-Vainicher, Iacopo Chiodini, Ivana Santi, Marco Massarotti, Luca Pietrogrande, Elisa Cairoli, Paolo Beck-Peccoz, Matteo Longhi, Valter Galmarini, Giorgio Gandolini, Maurizio Bevilacqua, Enzo Grossi

**Affiliations:** 1 Endocrinology and Diabetology Unit, Medical Sciences Department, Fondazione Istituto di Ricovero e Cura a Carattere Scientifico Cà Granda Ospedale Maggiore Policlinico, Milan, Italy; 2 Istituto Geriatrico Azienda di Servizi alla Persona Istituti Milanesi Martinitt e Stelline e Pio Albergo Trivulzio, Milan, Italy; 3 Istituto di Ricovero e Cura a Carattere Scientifico Humanitas, Rozzano, Milan, Italy; 4 Orthopedic Clinic, Dipartimento di Medicina, Chirurgia e Odontoiatria San Paolo, University of Milan, Milan, Italy; 5 Unit of Reumathology, Istituto di Ricovero e Cura a Carattere Scientifico Istituto Ortopedico Galeazzi, Milan, Italy; 6 Azienda Ospedaliera Fatebenefratelli e Oftalmico, Milan, Italy; 7 Istituto di Ricovero e Cura a Carattere Scientifico Don Gnocchi, Milan, Italy; 8 Endocrinology and Diabetes Unit, Luigi Sacco Hospital (Vialba), University of Milan, Milan, Italy; 9 Bracco Medical Department, San Donato Milanese, Milan, Italy; 10 Semeion Research Centre, Rome, Italy; Marienhospital Herne - University of Bochum, Germany

## Abstract

**Background:**

It is known that bone mineral density (BMD) predicts the fracture's risk only partially and the severity and number of vertebral fractures are predictive of subsequent osteoporotic fractures (OF). Spinal deformity index (SDI) integrates the severity and number of morphometric vertebral fractures. Nowadays, there is interest in developing algorithms that use traditional statistics for predicting OF. Some studies suggest their poor sensitivity. Artificial Neural Networks (ANNs) could represent an alternative. So far, no study investigated ANNs ability in predicting OF and SDI. The aim of the present study is to compare ANNs and Logistic Regression (LR) in recognising, on the basis of osteoporotic risk-factors and other clinical information, patients with SDI≥1 and SDI≥5 from those with SDI = 0.

**Methodology:**

We compared ANNs prognostic performance with that of LR in identifying SDI≥1/SDI≥5 in 372 women with postmenopausal-osteoporosis (SDI≥1, n = 176; SDI = 0, n = 196; SDI≥5, n = 51), using 45 variables (44 clinical parameters plus BMD). ANNs were allowed to choose relevant input data automatically (TWIST-system-Semeion). Among 45 variables, 17 and 25 were selected by TWIST-system-Semeion, in SDI≥1 *vs* SDI = 0 (first) and SDI≥5 *vs* SDI = 0 (second) analysis. In the first analysis sensitivity of LR and ANNs was 35.8% and 72.5%, specificity 76.5% and 78.5% and accuracy 56.2% and 75.5%, respectively. In the second analysis, sensitivity of LR and ANNs was 37.3% and 74.8%, specificity 90.3% and 87.8%, and accuracy 63.8% and 81.3%, respectively.

**Conclusions:**

ANNs showed a better performance in identifying both SDI≥1 and SDI≥5, with a higher sensitivity, suggesting its promising role in the development of algorithm for predicting OF.

## Introduction

Osteoporosis is a multi-factorial systemic skeletal disease, characterised by low bone mass and microarchitectural deterioration of bone tissue, with a consequent increase in bone fragility and susceptibility to fracture [1]. The diagnosis of osteoporosis relies on the measurement of bone mineral density (BMD), measured by dual energy X-ray absorptiometry (DXA), or on the presence of a fragility fracture. Nevertheless, the assessment of fracture risk with BMD shows several limitations. The bone microarchitecture, commonly named “bone quality”, is difficult to assess by clinical parameters [2], [3]. Also for this reason, the BMD detection rate for fragility fractures (sensitivity) is low, and the 96% of fragility fractures seems to arise in women without a densitometric diagnosis of osteoporosis [4]. The use of additional risk factors that, independently of BMD, add information on fracture risk, improves the BMD sensitivity in predicting fragility fracture [4], [5]. Thus, recent efforts by the World Health Organization Metabolic Bone Disease Group have focused on developing a risk assessment tool (FRAX™) using clinical risk factors with and without femoral neck BMD to enhance fracture prediction [6].

Asymptomatic morphometric vertebral fractures (MVF) are considered the “prima facies” of osteoporosis, and are much more prevalent than clinical fractures [7]. Morphometric vertebral fractures are often overlooked by radiologist [8], although they represent one of the strongest clinical predictors of subsequent fractures [9]. Indeed, the risk of subsequent fractures increases with both the number and the severity of prior vertebral fractures [10]–[13]. Thus, it may be more relevant for an appropriate assessment of future fracture risk to assess all the fractures rather than to consider the spine as a whole, as a binary parameter (fracture Yes/No), as in FRAX™. The spinal deformity index (SDI) described by Minne [14] and Genant [15] is an assessment tool that integrates both the number and severity of fractures by summing the vertebral fracture grades along the spine from T4 to L4. Two studies suggested that SDI may be an accurate tool for vertebral fracture prediction and that baseline SDI was predictive of the 3-year incidence of subsequent vertebral fracture [16], [17]. Recently, the SDI has been found to be negatively associated with functional outcome in women with hip fracture admitted consecutively to a rehabilitation hospital [18]. This is in line with histological data showing that microarchitectural deterioration is proportionally worse in women with increasing severity of vertebral fractures [19].

Recent studies suggest that FRAX™ tool may have a poor sensitivity for fracture prediction and does not significantly improve the discriminatory value of hip BMD alone [20], [21]. An explanation is that osteoporosis is a multi-factorial systemic skeletal disease, in which different factors and environments interact in stochastic, nonlinear biological mechanisms. Therefore, the link between bone mineral density, clinical risk factors and fragility fractures probably needs a special kind of mathematics, such as Artificial Neural Networks (ANNs), to be understood.

Artificial Neural Networks are artificial adaptive systems, inspired by the functioning processes of the human brain [22]. These mathematical informatics' systems are able to modify their internal structure in relation to a function objective. So, they are particularly suited for solving nonlinear problems, being able to reconstruct the fuzzy logic rules [23] that govern the optimal solution for these problems. The ability to learn through an adaptive way (i.e. extracting from the available data the information needed to gather a specific task and to generalise the acquired knowledge) is a characteristic that make the ANN models a very powerful tool for data analysis. The internal structure and functional organisation of such systems can be updated and modified with respect to the environmental changes enabling the ANN to create its own representation of the information. Moreover, these models are interesting also for their noise tolerance that allows accurate performances in presence of unreliability, wrong data or measurement errors.

Although ANNs offer promise for improving the predictive value of traditional statistical data analysis and have been successfully used in many areas of medicine [24], no reports have so far investigated the ability of ANNs in predicting osteoporosis fracture.

## Methods

### Objectives

The aim of the present study was to evaluate the capacity of ANNs, compared with Logistic Regression (LR), to recognise: 1) patients with or without morphometric vertebral fractures (SDI≥1 and SDI = 0 respectively) and 2) patients with SDI≥5 or without morphometric vertebral fractures (SDI≥5 and SDI = 0 respectively), on the basis of classical bone osteoporotic risk factors and other clinical information, routinely derived from the out-patient visits.

### Participants

The study population included 430 patients consequently referred to 9 out-patient clinics for osteoporosis management belonging to *GISMO-Lombardia Group* (North-West of Italy), from 1^st^ January to 31^st^ March 2010. The majority of the patients had been referred from primary care. The inclusion criteria were: 1) female post-menopausal patients with osteopenia or osteoporosis defined by the presence of a T-score for spine BMD or hip BMD ≤−1.0>−2.5 and ≤−2.5 respectively. Exclusion criteria were: 1) patients who have been treated with bisphosphonates or other drugs for osteoporosis, or who have been taking these drugs for at least 1 year; 2) secondary forms of osteoporosis; 3) malignancies; 4) renal failure; 4) previous or present treatment with oral corticosteroids or any other drug known to affect bone metabolism. Eventually, data from 372 subjects were analyzed.

Information on menopausal age, number of pregnancies, breast feeding, smoking habits, and alcohol consumption were collected. In order to estimate this latter variable, all subjects were asked about quantity and type of drinks consumed and data were converted as Units per day (8 gr of pure alcohol) [25]. Patients with alcohol consumption >100 gr per day were excluded, as possibly affected by a secondary form of osteoporosis (see the above-mentioned exclusion criteria). Moreover, family history of osteoporosis and of all type of hip fractures was obtained from all subjects at consultation. The patients were also asked about previous clinical fragility fractures at spine, ribs, wrist and hip. Fractures of skull, jaw, coccyx, phalanx, ankle, cervical and thoracic vertebrae (C1 to T4), and of posterior arches of the vertebra were not considered as osteoporosis-related fractures and were excluded from the analysis. In all patients, the presence of previous fragility fractures was ascertained by self report and no additional validation of this information was conducted.

In all patients height and weight were measured and body mass index (BMI) was calculated. Calcium intake, expressed as mg/day, was assessed using a simplified questionnaire described in a previous paper [26]. In particular, usual calcium intake coming from some selected calcium-rich foods (milk and dairy products) was estimated by a 7-day food frequency questionnaire. The foods checked include milk, aged cheese, soft cheese, cottage cheese, and yoghurt. Portion sizes were quantified by means of household measures (slices, cups, glasses). To standardize the slice weight, three cardboard samples of different size were used (about 100, 50 and 25 g). The number of standardized servings was assessed, each containing approximately 300 mg of calcium (a glass of milk, a cup of yoghurt, a portion of about 100 g of cottage cheese, a 50 g slice of soft cheese and a 25 g slice of aged cheese [26]. Patients were asked about co-morbidities (i.e. arterial hypertension, dyslipidemia, gastric/esophagus disease, anxiety, depression, chronic obstructive pulmonary disease (COPD), osteoarthritis, kidney stones, type 2 diabetes mellitus) (Table 1).

**Table 1:** Variables used in the analysis and variables selected by TWIST system in the subsequent analysis: SDI = 0 vs SDI≥1 (SDI≥1) and SDI = 0 vs SDI≥5 (SDI≥5).

	SDI≥1	SDI≥5
Age	x	
Age<68 years	x	x
Age≥68 years		x
Body Mass Index (BMI) Kg/m^2^	x	
BMI≤21	x	x
BMI>21<30		
BMI≥30	x	x
Years since menopause (YSM)	x	x
YSM<18		x
YSM≥18		x
Number of pregnancies		x
Months of breast feeding		
Current smoking yes	x	x
Current smoking no	x	
Previous smoking yes	x	
Previous smoking no		
Alcohol yes		x
Alcohol no		x
Bone mineral density T-score ≤−2.5 yes		
Bone mineral density T-score ≤−2.5 no		
Previous fragility fracture yes	x	x
Previous fragility fracture no		x
Familiar history of femoral fracture yes		x
Familiar history of femoral fracture no		
Calcium intake mg/day	x	x
Calcium intake ≤300 mg/day yes		x
Calcium intake ≤300 mg/day no	x	x
Arterial hypertension yes	x	
Arterial hypertension no		x
Dyslipidemia yes		
Dyslipidemia no	x	x
Gastric/oesophagus disease yes		x
Gastric/oesophagus disease no		x
Anxiety/depression yes	x	x
Anxiety/depression no		x
Chronic pulmonary obstructive disease (COPD) yes		
Chronic pulmonary obstructive disease (COPD) no		x
Osteoarthritis yes		
Osteoarthritis no		
History of kidney stones yes	x	
History of kidney stones no		
Type 2 Diabetes Mellitus (T2D) yes	x	x
Type 2 Diabetes Mellitus (T2D) no		
SDI = 0		
SDI≥1		
SDI≥5		

SDI≥1: Variables selected by TWIST system in the analysis aimed to differentiate patients with SDI≥1 from those with SDI = 0 (the number 17, reported in Table 4a, refers to a maximisation of these variables); SDI≥5: Variables selected by TWIST system in the analysis aimed to differentiate patients with SDI≥5 from those with SDI = 0 (the number 25, reported in Table 4b, refers to a maximisation of these variables).

Twist system can easily select just one of the two binary forms of the variables since that choosing one option implies also the information of its complement.

Since ANNs cannot analyze missing values, patients who missed one or more information were excluded. Eventually, 372 post-menopausal patients were considered for the analysis.

### Description of investigations undertaken

Dual energy X-ray absorptiometry (DXA) scans were carried out to measure BMD at the spine and hip with the instrument available locally: Hologic Discovery (Watham MA, USA) in 73% and Lunar GE (Lunar Pty Ltd., Madison, Wisconsin, USA) in 27% of the centres. All patients had at least one measurement among hip or spine and were classified as having or not a low BMD (T-score > or ≤−2.5 at least at one site, respectively). No cross-calibration was undertaken between the Hologic and Lunar machines.

Conventional spinal radiographs in lateral (T5–L4) and anterior-posterior (AP) projection (L1–L4) were obtained in all subjects with standardised technique. The vertebrae were identified and evaluated by the clinicians of the osteoporosis centres by using dedicated software for quantitative morphometry (MorphoXpress, Optasia Medical, Warner Chilcott, Rockaway, NJ, USA) [27]. In brief the MorphoXpress operates as follows: original lateral vertebral radiographs are digitised using a TWAIN scanner (UMAX Power Look 1000, Techville, Dallas, TX, USA). Analysis is then initialised by the manual targeting of the centres of the upper and lower vertebrae to be analysed. The software then automatically finds the positions of landmarks for a standard 6-point morphometry measurement. The software then allows these points to be moved by the operator, if deemed necessary, before the points are confirmed as being correct. The positions of the confirmed points are then used by the software to calculate anterior, middle and posterior vertebral heights, which may also be used for the determination of deformity shape [27]. Before beginning the study in each participating centre, adequate training was given to a single operator in order to standardise the use of quantitative morphometry. In each centre, the operator read at least 15 radiographs more than once to assess how reliable within himself he was. In order to assess the correct identification of thoracic vertebral fractures, copies of the X-rays from 50 patients randomly taken from centres were sent to an experienced radiologist. Inter-reader reliability between results obtained in various centres and central assessment by the experienced radiologist, summarised by the kappa statistics (k) test, was 0.85. The fractures were defined as intact (SQ grade 0) or as having approximately mild (20–25% compression), moderate (25–40% compression), or severe (>40% compression) deformity (SQ grades 1, 2 and 3, respectively). Subsequently, for each subject the spinal deformity index (SDI) was calculated by summing the SQ grade for each of the 13 vertebrae from T5 to L4 (SDI = SQT4+…+SQT12+SQL1+…+SQL4) (17).

### Ethics

Ethics Committee approval was not required since data were collected as part of the standard care for the patients and the data sets available to researchers were fully de-identified.

### Statistical methods

The 9 out-patient clinics for osteoporosis management belonging to GISMO-Lombardia Group have been working altogether since September 2005 using the same protocols for data collection. The homogeneity between the 9 centres has been checked by specific investigators meetings twice yearly, but a Inter-rater Cohen's k of agreement was not obtained.

We performed the analysis, both the traditional and ANNs, using two different end-points: 1) SDI≥1 (This identifies eligibility for full reimbursement for osteoporosis treatment according to the Italian rules: “Nota 79”) [28]; 2) SDI≥5. This latter end-point was chosen considering that there is an almost linear relationship between SDI and fracture risk, not influenced by the particular fracture configurations, till a SDI of 5 [16].

### Logistic Regression Analysis

Statistical analysis was performed by SPSS version 12.0 statistical package (SPSS Inc., Chicago, IL, USA). The normality of distribution was checked by Kolmogorov-Smirnov test. The results are expressed as mean±SD if not differently specified. Comparison of continuous variables between groups was performed using Student's t-test or Mann-Whitney test on the basis of the normality of distribution. Categorical variables between the two groups were compared by χ^2^test. The bivariate associations between SDI and all the variables were tested by Pearson product moment association or Spearman correlation as appropriate. In all patients, logistic regression analysis assessed the association between the presence of SDI≥1 or SDI≥5 as dependent variables (expressed as categorical variables) and the presence of previous fragility fracture, arterial hypertension, chronic obstructive pulmonary disease, low BMD (T-score ≤−2.5) and dyslipidemia (independent variables, expressed as categorical variables) and years since menopause and daily calcium intake (independent variables, expressed as continuous variables). We decided to include in the logistic regression model those variables, which were found to be different between fractured and not fractured subjects.

Bone mineral density data were categorized on the basis of a T-score > or ≤2.5, which is the commonly used threshold to define osteoporosis [1], [2], to avoid the possible influence of having used two different instruments for determining BMD.

The alcohol consumption was collected as a categorical variable (presence of alcohol consumption < or ≥3 alcohol units/day), as persons consuming more than 3 alcohol units per day have been demonstrated to have an higher risk of fractures compared to abstainers, while a precise range of beneficial alcohol consumption has not been determined so far, although available evidence suggest a favourable effect of 0.9–1.8 alcohol units per day [29].

We employed a classical multivariable logistic regression including all variables and then building the final model using forward stepwise logistic regression. A significance level of 0.3 was required to allow a variable into the model, and a significance level of 0.35 was required for a variable to stay in the model. In addition, the data set has been re-analyzed also building a multiple model including in the logistic regression analysis only the variables with p<0.25 in bivariate analysis. P values of ≤0.05 were considered significant.

### Artificial neural networks analysis

Advanced intelligent systems based on novel coupling of artificial neural networks and evolutionary algorithms have been applied. In this study we applied TWIST system and supervised ANNs in order to develop a model able to predict with a high degree of accuracy the diagnostic class starting from available data. Supervised ANNs are networks which learn by examples, calculating an error function during the training phase and adjusting the connection strengths in order to minimize the error function. [30]. The learning constraint of the supervised ANNs makes their own output coincide with the predefined target. The general form of these ANNs is: y = f(x,w*), where w* constitutes the set of parameters which best approximate the function.

The trained ANN generates a single output which is a continuous variable that can range between 0 and 1. However, as our ‘real’ dependent variables are binary (0 or 1), the ANN output needs to be reduced to 0 or 1 using a specific threshold. If the ANN gives as output values from 0 to 0.5 then the output is considered as 0 (e.g SD 5 absent therefore SD 1); while is the output is comprised from 0.51 to 1 then the output is considered as 1(e.g SD 5 present therefore SD 5). The ROC curve, which measures sensitivity and 1-specificity (the false positive rate) across different cutoffs, is then generated varying the threshold for binary classification.

Data analysis was performed using a re-sampling system named TWIST developed by Semeion Research Centre. The TWIST system consists in an ensemble of two previously described systems: Training & Testing (T&T) and Input Selection (I.S) [31]. The T&T system is a robust data re-sampling technique that is able to arrange the source sample into sub-samples that all possess a similar probability density function. In this way, the data is split into two or more sub-samples in order to train, test and validate the ANN models more effectively. The IS system is an evolutionary wrapper system able to reduce the amount of data while conserving the largest amount of information available in the dataset. The combined action of these two systems allow us to solve two frequent problems in managing Artificial Neural Networks, i.e. the optimal splitting of the data set in training and testing subsets containing a balanced distribution of outliers and the optimal selection of variables with maximal amount of information relevant to the problem under investigation. Both systems are based on a Genetic Algorithm, the Genetic Doping Algorithm (GenD) developed at Semeion Research Centre [32], [33]. After this processing, the features that were most significant for the classification were selected and at the same time the training set and the testing set were created with a function of probability distribution similar to the one that provided the best results in the classification. Twin supervised Multi Layer Perceptron, with four hidden units, were then used for the classification task employing a crossover training-testing procedure (named a–b; b–a, where the a-subset first function as training data and b-subset as testing data, then they are reversed) The twin ANNs which were trained and tested on the new data set generated by TWIST systems are “virgin” and operate independently and blindly from each other and from TWIST system.

The validation protocol is a procedure to verify the models' ability to generalize the results reached in the testing phase. Among the different protocols reported in literature, the selected model is the protocol with the greatest generalization ability on data unknown to the model itself. The procedural steps in developing the validation protocol are: 1) subdividing the dataset randomly into two sub-samples: the first called Training Set, and the second, called Testing Set; 2) choosing a fixed ANNs (and/or Organism) which is trained on the Training Set. In this phase, the ANNs learns to associate the input variables with those that are indicated as targets; 3) saving the weight matrix produced by the ANNs at the end of the training phase, and freezing it with all of the parameters used for the training; 4) showing the Testing Set to the ANNs, so that in each case, the ANNs can express an evaluation based on the training just performed. This procedure takes place for each input vector but every result (output vector) is not communicated to the ANNs; in this way, the ANNs is evaluated only in reference to the generalization ability that it has acquired during the Training phase; 5) constructing a new ANNs with identical architecture to the previous one and repeating the procedure from point 1. This protocol is applied once starting from the first subsample and once starting from the second subsample taken as training set obtaining in this way 2 independent classification experiments. This procedure was repeated 10 times according to 5×2 cross-validation protocol [34]. In this procedure the study sample is five-time randomly divided into two sub-samples, always different but containing similar distribution of cases and controls. Training and testing sets are then reversed and consequently 10 independent models carried out [34].

We estimated for each strategy sensitivity, specificity, and overall accuracy. We also calculated the areas under the receiver operating curve (ROC) in an empirical (non-parametric) approach, which were used for comparison among the different strategies [35]. The statistical comparison among ROC curves was performed as described elsewhere [36]. The ROC represents the relationship between sensitivity and specificity for the prediction of each of the considered outcomes.

Differences were considered significant at a 5% probability level.

## Results

Clinical characteristics of patients (all, SDI 0, SDI≥1 and SDI≥5) are reported in Table 2. The patients with SDI≥1 were older and farer from menopause than patients with SDI = 0. Moreover, they showed a higher prevalence of arterial hypertension and a lower prevalence of anxiety/depression in respect with SDI = 0 patients. Similarly, as compared with SDI = 0 patients, SDI≥5 subjects were older, farer from menopause, and had a higher prevalence of calcium intake ≤300 mg/day, arterial hypertension, previous fragility fractures and COPD and a lower prevalence of dyslipidemia.

**Table 2:** Clinical characteristics of all patients, patients without morphometric vertebral fractures, SDI≥1 and SDI≥5.

	All (n = 372)	SDI = 0 (n = 196)	SDI≥1 (n = 176)	SDI≥5 (n = 51)	*P	#P
**Age** (years)	68.0±8.5	65.3±8.1	71.1±7.8	75.2±6.1	0.0001	0.0001
**YSM** (years)	18 (1–50)	16 (1–44)	22.5 (3–50)	27 (8–50)	0.0001	0.0001
**BMI** (Kg/m^2^)	23.0 (16–41)	23 (16–41)	23 (16–36)	24 (16–36)	0.374	0.160
**Calcium intake** (mg/day)	636±404	640±382	632±429	596±458	0.861	0.486
**n. of pts with calcium intake≤300 mg/day** (%)	73 (19.6)	32 (16.3)	41 (23.3)	16 (31.4)	0.116	0.027
**n. of pregnancies**	2 (0–5)	2 (0–5)	2 (0–4)	2 (0–4)	0.795	0.383
**BF months**	3 (0–72)	3 (0–60)	3 (0–72)	3 (0–72)	0.255	0.352
**n. of smokers** (%)	57 (15.3)	28 (14.3)	29 (16.5)	5 (9.8)	0.568	0.494
**n. of ex-smokers** (%)	38 (10.2)	22 (11.2)	16 (9.1)	4 (7.8)	0.608	0.613
**n. of patients consuming alcohol ≥3 units/day** (%)	124 (33.3)	63 (32.1)	61 (34.7)	19 (37.3)	0.660	0.507
**n. of patients with previous clinical fracture** (%)	33 (8.9)	14 (7.1)	19 (10.8)	10 (19.6)	0.273	0.014
**n. of patients with familiar history of hip fracture** (%)	64 (17.2)	35 (17.9)	29 (16.5)	9 (17.6)	0.784	1.000
**n. of patients with kidney stones** (%)	17 (4.6)	10 (5.1)	7 (4.0)	1 (2.0)	0.630	0.468
**n. of patients with arterial hypertension** (%)	110 (29.6)	47 (24.0)	63 (35.8)	20 (39.2)	0.017	0.035
**n. of patients with dyslipidemia** (%)	55 (14.8)	35 (17.9)	20 (11.4)	2 (3.9)	0.081	0.014
**n. of patients with gastric/esophagus disease** (%)	84 (22.6)	47 (24.0)	37 (21.0)	8 (15.7)	0.536	0.258
**n. of patients with anxiety/depression** (%)	50 (13.4)	33 (16.8)	17 (9.7)	9 (17.6)	0.048	0.837
**n. of patients with COPD** (%)	14 (3.8)	4 (2.0)	10 (5.7)	5 (9.8)	0.099	0.020
**n. of patients with Osteoarthritis** (%)	80 (21.5)	46 (23.5)	34 (19.3)	11 (21.6)	0.377	0.854
**n. of patients with T2D** (%)	14 (3.8)	8 (4.3)	6 (3.4)	1 (2.0)	0.791	0.690
**n. of patients with Low BMD (T-score ≤−2.5)** (%)	242 (65.1)	127 (64.8)	115 (65.3)	35 (68.6)	1.000	0.741
**SDI**	0 (0–24)	0 (0–0)	2 (1–24)	8 (5–24)		

Data are expressed as mean±SD, and median (range) for not normally distributed variables, if not differently specified.

*SDI = 0 vs SDI≥1; #SDI = 0 vs SDI≥5; SDI: Spinal Deformity Index; YSM: Years since menopause; BMI: Body Mass Index: weight (Kg)/height ^2^ (m^2^); BF: breast feeding expressed in months; COPD: chronic obstructive pulmonary disease; T2D: Type 2 diabetes mellitus; SDI: Spinal Deformity Index calculated according to the method described by Crans (see Methods);

The linear correlation index between the input variables and the Spinal Deformity Index was generally very low (range R: 0.000–0.384). The SDI was significantly and positively associated with age (*R* = 0.374, *p* = 0.0001), and with years since menopause (YSM) (*R* = 0.384, *p* = 0.0001). Logistic regression analysis showed that YSM and the absence of dyslipidemia were independently associated with SDI≥1, regardless for the presence of low BMD (Table 3A). Moreover, this analysis showed that YSM, COPD and the absence of dyslipidemia were independently associated (and the presence of a previous fragility fracture borderline associated) with the presence of SDI≥5, regardless of low BMD (Table 3B).

**Table 3:** OR for detecting morphometric vertebral fracture (SDI≥: A and SDI≥5: B) for Potential Risk Factors using the multivariable Logistic Regression Model.

	OR	95% CI	p
**A**			
**Years since menopause**	1.07	1.04–1.09	0.0001
**Previous fragility fracture**	1.28	0.60–2.75	0.522
**Arterial Hypertension**	1.54	0.93–2.55	0.093
**COPD**	2.63	0.74–9.31	0.134
**Daily calcium intake (mg/day)**	1.00	1.00–1.00	0.664
**Low BMD (T-score ≤−2.5)**	1.06	0.67–1.67	0.811
**Dyslipidemia (absence)**	2.21	1.15–4.24	0.017
**B**			
**Years since menopause**	1.13	1.08–1.19	0.0001
**Previous fragility fracture**	2.93	0.98–8.75	0.054
**Arterial Hypertension**	1.81	0.80–4.11	0.154
**COPD**	7.11	1.12–45.19	0.038
**Daily calcium intake (mg/day)**	1.00	1.00–1.00	0.134
**Low BMD (T-score ≤−2.5)**	1.22	0.55–2.73	0.629
**Dyslipidemia (absence)**	12.5	2.21–71.43	0.004

The variables selected by TWIST system, at the end of its evolution, for the analysis aimed to identify patients with a SDI≥1 and a SDI≥5, are reported in Table 1.

**Figure 1:**
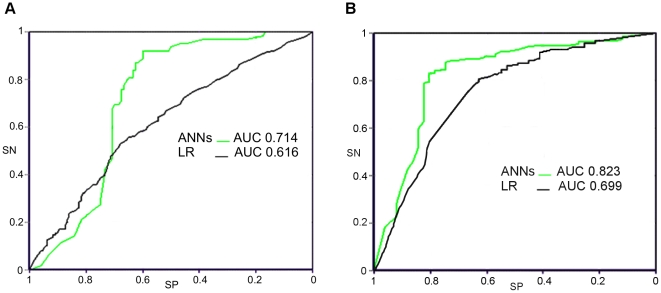
ROC curve for artificial neural networks and logistic regression analysis in identifying SDI≥1 and SDI≥5. The ANN AUC is significantly superior to LR AUC both in identifying SDI≥1 (p<0.01) (A) and SDI≥5 (p<0.001) (B). ROC: Receiver operating characteristic, SN: sensitivity, SP: specificity. ANNs: artificial neural networks; AUC: area under the curve; LR: logistic regression analysis.

The sensitivity, specificity and accuracy of ANNs and LR (by Forward Stepwise method) in discriminating patients with SDI = 0 from those with SDI≥1 and from those with SDI>5 are reported in table 4A and 4B, respectively. The sensitivity, specificity and accuracy of ANNs represent the mean of the ten experiments for each target prediction according to five per two cross-validation protocol (tables 5A and 5B) [34]. In both the analysis the overall accuracy of ANNs as evaluated with ROC AUC was significantly superior to that of LR (table 4A and 4B and figures 1a and 1b).

**Table 4:** Sensitivity, Specificity and overall accuracy in identifying patients with a SDI≥1 (A) and SDI≥5 (B) by artificial neural networks analysis and traditional statistics.

	n° of variables	SN (%)	SP (%)	Accuracy (%)	ROC AUC
		(95% CI)	(95% CI)	(95% CI)	(95%CI)
**A**					
**ANNs**	17	72.5 (65.91–79.09)	78.5 (72.75–84.00)	75.5 (71.13–79.87)	0.714** (0.673–0.755)
**LR**	45	35.8 (30.22–44.38)	76.5 (70.40–83.61)	56.2 (51.78–60.62)	0.616 (0.576–0.656)
**B**					
**ANNs**	25	74.8 (62.89–80.72)	87.8 (83.22–92.38)	81.3 (76.44–86.16)	0.823** (0.780–0.866)
**LR**	45	37.3 (25.14–49.46)	90.3 (86.16–94.44)	63.8 (57.88–69.72)	0.699 (0.657–0.741)

ANNs: artificial neural networks; LR: logistic regression analysis; SN: sensitivity; SP: specificity; ROC: receiver operating characteristic; AUC: area under the curve.

**: p<0.01.

**Table 5:** Goodness of fit test for ANNs in identifying patients with a SDI≥1 (A) and SDI≥5 (B).

	Testing on subset	Sensitivity (%)	Specificity (%)	Overall accuracy (%)
**A**	**1a**	73.4	78.9	76.2
	**1b**	71.7	79.2	75.5
	**2a**	72.0	77.5	74.8
	**2b**	73.2	77.9	75.6
	**3a**	74.0	78.0	76.0
	**3b**	71.2	79.2	75.2
	**4a**	72.3	77.9	75.1
	**4b**	72.9	78.7	75.8
	**5a**	72.2	78.8	75.5
	**5b**	72.1	78.9	75.5
	**Average**	72.5	78.5	75.5
**B**	**1a**	77.3	87.9	82.6
	**1b**	72.4	87.6	80.0
	**2a**	73.2	88.4	80.8
	**2b**	74.5	88.2	81.4
	**3a**	74.5	87.6	81.1
	**3b**	74.2	87.5	80.9
	**4a**	76.6	87.8	82.2
	**4b**	78.0	88.2	83.1
	**5a**	75.4	87.3	81.4
	**5b**	72.3	87.3	79.8
	**Average**	74.8	87.8	81.3

5×2 cross validation protocol.

A: Chi square = 0.10; N.S.; B: Chi square = 0.23; N.S.

The analysis performed by a multiple model including in LR only the variables with p<0.25 in bivariate analysis, showed less satisfactory results than those obtained with the Forward Stepwise method (data not shown).

## Discussion

The present study showed that Artificial Neural Networks have a better capacity of discriminating between patients without morphometric vertebral fractures (SDI = 0) and patients with at least 1 morphometric vertebral fractures (SDI≥1) and patients with a SDI≥5, than logistic regression analysis. Moreover, in our sample, the LR showed a low sensitivity in identifying SDI≥1 and SDI≥5. This result is in keeping with some previous studies suggesting that the algorithm using classical statistical approach, like FRAX™, have a low sensitivity [20], [21], probably due to the limitations of a linear analytical approach in explaining a complex multifactorial disease, like osteoporosis.

To our knowledge, this is the first study which aimed to evaluate the capacity of ANNs, compared with Logistic Regression (LR), to recognise patients with fragility vertebral fractures. The present results are similar to those obtained in previous studies in other medicine fields [37]–[42], suggesting that this kind of statistical approach may be better than the traditional one, to understand complex topics like human diseases.

The comparison of results obtained with two different analytical approaches (logistic regression and TWIST system), points out the need to employ systems that are really able to handle the disease complexity instead of treating the data with reductionist approaches that are unable to detect multiple contribution of smaller effect in predisposing to the disease. Moreover, ANNs are able to identify variables combinations that are likely to produce accurate predictions of outcomes for a single individual, a very important property for the clinician facing every day with decision to be taken in a specific patient. The superiority of ANNs vs LR may be also due to the fact that ANNs build up models with higher number of variables, since they can manage also variables with a very poor linear correlation index. Another novelty of this study is the possibility of identifying the presence of a morphometric vertebral fracture (SDI≥1) as a binary parameter (fracture Yes/No), but also the presence of a high SDI (SDI≥5), that is known to be associated with a higher risk of new vertebral fractures [16], regardless for BMD. This is an important point since it has been demonstrated that also the risks of hip and any non vertebral fractures increase with SDI [43]. Thus, the use of SDI combined with other risk factors, particularly if with an ANNs algorithm, may consent to better identify patients at high risk of fractures. It must be considered that an histomorphometric study showed that the SDI is inversely associated with bone volume, therefore suggesting SDI as a surrogate marker of bone quality [19].

The presence of a fragility fracture was found to be associated with hypertension by the ANNs analyses (tending to the statistical significance also by LR analysis) and with the absence of dyslipidemia by both the LR and ANNs analyses (Table 3A). This result, although not a declared end-point deserves particular interest. Indeed, the link between arterial hypertension, BMD and fractures is debated and may be possibly explained by the use of antihypertensive drugs. Several studies showed an association between the use of antihypertensive drugs, such as diuretic loops, and BMD loss, probably due to an increase in urinary calcium excretion, but the effect on fracture risk is still controversial [44], [45]. On the other hand, another study suggested that there was no association between BMD and hypertension after correction for several confounding factors [46]. Recently, the sensitivity to glucocorticoids has been suggested as a possible link between hypertension and osteoporosis. Indeed, the combination of hypertension and vertebral fractures has been shown to be associated with the sensitizing polymorphisms of the glucocorticoid receptor in patients with a subtle cortisol excess [47].

Similarly, the association between the dyslipidemia and fractures risk probably has a reference to the use of statins. Statins inhibits cholesterol synthesis by blocking the initial part of the mevalonate metabolic pathway, which is the same metabolic pathway inhibited, more downstream, by bisphosphonates. Moreover, lipophilic statins seem to influence bone formation influencing the expression of bone morphogenetic protein 2 (BMP-2) [48]. Finally, in several clinical trials the use of statins had been associated with a reduction in the risk of fracture [49], [50].

The relatively small sample size is a limitation of the present study. However, as the prevalence of morphometric vertebral fractures is higher than that of clinical fractures, the use of the formers as end point, allows reducing the sample size saving the statistic power of the study. It is well known that ANNs, at variance with the classical statistical tests, can manage complexity even with relatively small samples and the subsequent unbalanced ratio between variables and records. Indeed, it is important to note that adaptive learning algorithms of inference, based on the principle of a functional estimation like artificial neural networks, can partially overcome the problem of dimensionality. Moreover, the cross-sectional design of the study does not consent to look at the incidence of fractures and to compare, head to head, this approach with FRAX. Another limit of the study may be related to the lack of a cross-calibration of different devices for measuring BMD. However, this possible error should have been corrected, at least in part, by the use of the T-score for expressing bone mineral density data and by having dichotomized the variable on the basis of a T-score > or ≤2.5. Similarly, it must be note that a inter-rater Cohen's k of agreement was not obtained between results obtained in various centers. However, the 9 out-patient clinics for osteoporosis management belonging to GISMO-Lombardia Group have been working altogether since September 2005 using the same protocols for data collection, and the homogeneity between the 9 centers has been checked by specific investigators meetings twice yearly. Finally, we do not have information about pharmacological history other than anti-osteoporotic drugs. It must be also considered that the study was conducted in female osteoporosis clinic patients, and, therefore, the extrapolation of the results to other groups should be done with caution. In the future, wider longitudinal studies could help to confirm our data and to better understand causal relationship between variables.

Notwithstanding these limitations, the present study shows, for the first time, that ANNs have a better capacity, in respect with LR, in identifying the presence of morphometric vertebral fractures and the presence of a high risk SDI. The use of ANNs in developing algorithms for predicting the fracture risk in the individual subject may be an important advance in assessing the most cost-effective therapeutic threshold in the field of osteoporosis.
